# RUNX1/miR-582-5p Pathway Regulates the Tumor Progression in Clear Cell Renal Cell Carcinoma by Targeting COL5A1

**DOI:** 10.3389/fonc.2021.610992

**Published:** 2021-04-14

**Authors:** Jianxin Xue, Shenhao Zhu, Feng Qi, Kai Zhu, Pu Cao, Jie Yang, Zengjun Wang

**Affiliations:** ^1^ Department of Urology, The Second Hospital of Nanjing, Nanjing University of Chinese Medicine, Nanjing, China; ^2^ Department of Urology, The First Affiliated Hospital of Nanjing Medical University, Nanjing, China; ^3^ Department of Urology, Jiangsu Cancer Hospital, Nanjing Medical University, Nanjing, China

**Keywords:** ccRCC, miR-582-5p, Runx1, proliferation, invasion

## Abstract

Recent evidences indicated that miRNAs played core role in the progression of clear cell renal cell carcinoma (ccRCC). However, its molecular mechanism in ccRCC is still remained unclear. The study was designed to identify the role and regulatory mechanism of miR-582-5p in ccRCC. In this study, the low expression level of miR-582-5p were detected by qRT-PCR in ccRCC patient tumor samples and ccRCC cell lines, respectively. The expression level of miR-582-5p was associated with tumor stage and metastasis. *In vivo* and *in vitro* experiments found miR-582-5p inhibit tumor growth *via* suppressing COL5A1 expression. Additionally, RUNX1 was identified as the negative regulator of miR-582-5p through database prediction and chromatin immunoprecipitation. Finally, the negative relation of RUNX1 and miR-582-5p was verified through rescue experiment both *in vitro* and *in vivo*. In summary, miR-582-5p, which was regulated by RUNX1, inhibited tumor growth and invasion by targeting COL5A1, indicating that miR-582-5p may act as a biomarker and that the RUNX1/miR-582-5p/COL5A1 axis could be a potential therapeutic target for ccRCC.

## Introduction

Renal cell carcinoma (RCC) is one of the most common and lethal malignancy with a global estimate for 403,262 new cases and 175,098 deaths according to the American Cancer Society (1). RCC contains three major histological subtypes clear cell RCC (ccRCC), papillary RCC (pRCC) and chromophobe RCC (chRCC) (2). ccRCC is the most common one which accounts for 75 percent among all RCC cases (3). Over the years, the incidence of RCC is gradually increasing probably due to an increase in the incidental detection of renal masses. In addition, although most of new diagnosed RCC tumor are localized, there are considerable cases with locally advanced diseases or metastases (4, 5). Hence, exploring the accurate factors and mechanisms responsible for tumorigenesis or development is the main goal, which may be valuable for early diagnosis and outcome predicting (6).

MicroRNAs are single-stranded non-coding RNAs containing approximately 23 nucleotides. They function as regulator of gene expression through binding to the 3’ untranslated region (3’UTR) of selected messenger RNA (mRNA). As a consequence, they induce either degradation or translational repression of mRNAs (7, 8). Previous evidences demonstrated that miRNAs are involved in several biological processes, such as cell growth, apoptosis, differentiation, and tumor tumorigenesis (9–12). As a member of miRNAs family, numerous studies showed that miR-582-5p was critical to the development of several solid tumors, including glioblastoma, prostate cancer, gastric cancer, lung cancer, oral cancer, colorectal cancer, bladder cancer, salivary adenoid cystic carcinoma, hepatocellular carcinoma, etc. (13–23). Unexpectedly, although a majority of studies verified that miR-582-5p is a tumor suppressor, other studies held different opinions. For instance, Maeno A et al. proved that miR-582-5p contributed to an increase in the proliferation of prostate cancer cells under androgen deprived conditions (20). Similar conclusion was draw by Shu Z et al. that miR-582-5p induced progression of colorectal cancer (17). Considering these controversial results, we were interested in the role of miR-582-5p in RCC, which still remained unclear.

In the present study, we showed that miR-582-5p was down-regulated in ccRCC cell lines and tissues, and over-expression of miR-582-5p suppressed the growth and migration *in vitro* and *in vivo*. Interestingly, our findings indicated that miR-582-5p, which was mediated by RUNX1, accelerated ccRCC progression through promoting the expression of COL5A1.

## Materials and Methods

### Tissue Specimens

From April to August 2019, 40 pairs of ccRCC tissues and their adjacent normal tissues were collected from patients following radical nephrectomy at Jiangsu Province Hospital, First Affiliated Hospital of Nanjing Medical University. The project was approved by the Ethics Committee of author’s institution. All procedures on the enrolled patients were informed in advance with written consent. All fresh samples were gathered after surgery and frozen in liquid nitrogen immediately and then stored at -80°C refrigerator until RNA extraction.

### Cell Culture and Transfection

Human renal proximal tubule epithelial cell line HK-2 and human ccRCC cell lines 786-O and Caki-1 were purchased from the Chinese Academy of Sciences (Shanghai, China). HK-2 cells were cultured in DMEM-F12k medium (Thermo Fisher Scientific, Waltham, MA, USA), and other cells were cultured in RPMI-1640 medium (Thermo Fisher Scientific) supplemented with 10% FBS (TransGen Biotech, Beijing, China), 100 U/mL penicillin, and 100 µg/mL streptomycin (Solarbio, Beijing, China). Cells were maintained in a humidified incubator at 37°C in 5% CO2.

Besides, selected cell lines were transfected with pcDNA3.1-COL5A1 and pcDNA3.1-RUNX1 (GenePharma, Shanghai, China). The mimics for miR-582-5p was also obtained from GenePharma. Transfection of these into cells was performed with Lipofectamine 2000 (Invitogen; Thermo Fisher Scientific, Waltham, MA, USA) following the manufacturer’s protocol.

### RNA Extraction and Quantitative Real-Time PCR (qRT-PCR)

Total RNA was isolated from tissues and cells using TRIzol Reagent (Invitrogen, CA, USA). cDNA was synthesized from 2μg of total RNA using a SuperScript II Reverse Transcriptase kit(Invitrogen). qRT-PCR was performed using an the Power SYBR Green Master Mix (Applied Biosystems). The comparative threshold cycle (CT) (2^-ΔΔCt^) method was used to analysis the relative fold changes in gene expression. The U6 and GAPDH were used as internal controls.

### Protein Isolation and Western Blot Assay

Proteins were extracted from the cultured cells with RIPA lysis buffer (1% NP40, 0.1% sodium dodecyl sulfate (SDS), 100 μg/ml phenylmethyl sulfonyl fluoride, and 0.5% sodium deoxycholate, in PBS) on ice. The supernatants were collected after centrifugation at12000×g at 4°C for 20min. After the protein concentration was determined using a BCA protein assay kit (Bio-Rad, China), the lysates were mixed with 4 × SDS loading buffer (125 mmol/l Tris-HCl, 4% SDS, 20% glycerol, 100 mmol/l DTT, and 0.2% bromophenol blue) at a ratio of 1:3. After the samples were heated at 100°C for 5 min proteins were differentiated on SDS polyacrylamide gels. The separated proteins were then transferred to a PVDF membrane. The membrane blots were probed with a primary antibody of rabbit IgG anti-COL5A1 (1:1,000) or rabbit IgG anti-glyceraldehyde 3-phosphate dehydrogenase (1:1,000) (Cell Signaling Technology Inc., Boston, MA, USA) and horseradish peroxidase-conjugated anti-rabbit IgG (Jackson ImmunoResearch Labs, West Grove, PA, USA) secondary antibody, and developed with the enhanced chemiluminescent system. The signals were recorded using X-ray film. GAPDH was used as an internal reference for protein COL5A1 and RUNX1.

### Cell Proliferation Assay

The proliferation ability of cells was evaluated using the Cell Counting Kit-8 (CCK-8, Dojindo, Kumamoto, Japan) after transfection. Twenty-four hours after transfection, cells were planted into 96-well plates (3x103 cells/well). For concentration gradient assay, 10µL of CCK-8 reagent was added into each well of the 96-well plates after incubation for 48 h, while being added at various time points (24h, 48h, 72h and 96h) for time gradient assay. Then the 96-well plates were placed at 37 °C for 2h, and the absorbance at 450 nm was measured using Infinite F50^®^ microplate reader (Tecan, CH), Subsequently. Each experiment was carried out in four-replicate wells and repeated three times.

### Colony Formation Assay

48 hours after cell transfection, 786-O or Caki-1 cells (1 x 10^3^ cells per well) was seeded into a six-well plate for 14 days. After fixed with 10% formaldehyde for 20 min, the forming colonies were then stained with 0.1% crystal violet (Sigma-Aldrich Co.). Then the number of foci >100 cells was counted.

### Cell Invasion Assay

Transwell assay was carried out to explore the invasive and migratory capacities of ccRCC cells with 8-µm pore sized transwell chamber (BD Biosciences, San Jose, CA, USA). For invasion assay, the upper chambers were precoated with a matrigel (BD Biosciences). The transfected cells at 5×10^3^ cells/well resuspended in FBS-free medium were plated into the top chamber. Medium containing 10% FBS was added to the bottom chambers. After incubated for 48 hrs, the noninvading or migrating cells were scraped whereas cells adhering to the bottom surface of the upper chamber were fixed and stained. Finally, the numbers of cells were calculated by counting five random fields under the microscope (Olympus). All assays were performed in triplicate.

### Xenograft Model

Murine xenografts were established in 6- to 8-week-old male nude mice (BALB/c-null) using 786-O or Caki-1 cells. A total volume of 150μl containing 2.5×106 tumor cells were inoculated subcutaneously into the backs. Each group consisted of 5 animals. Tumor growth was monitored every 4 days in each group. Tumor volume was calculated (width²×length)/2 at each time point. The mice were sacrificed 28 days later, and the tumors were photographed and stained with hematoxylin/eosin. All animal studies were approved by the animal ethics committee of Nanjing Medical University.

### Immunohistochemistry (IHC)

All the extracted samples were fixed with formalin (4%) and embedded in paraffin. Mice tumor tissues were sliced into tissue sections (5 μm thickness) and incubated with antibodies for Ki-67 (Abcam, ab92742) overnight at 4 °C. The sections were incubated with the secondary HRP Goat Anti-Rabbit IgG (Abcam, ab205718) at room temperature for 1 h, and then were stained with DAB solution. The nuclei were counterstained with hematoxylin. An Olympus microscope (Olympus, Tokyo, Japan) was used to evaluate the images.

### Mammalian Lentiviral Overexpression Plasmid Construction

The coding sequence of COL5A1 and RUNX1 was amplified and cloned into pLEX-MCS, and the pLEX-GFP was used as a control. To generate lentiviral particles, plasmids were co-transfected into HEK293T cells along with envelope (VSVG) and packaging (Delta 8.9) plasmids using Lipofectamine 2000 (Invitrogen). The viral supernatants were harvested and filtered after two days of transfection. Cells were infected with 8 μg/ml polybrene. Overexpression efficiencies were confirmed *via* western blotting.

### Bioinformatics Database Analysis

An online tool dbDEMC 2.0 (https://www.picb.ac.cn/dbDEMC/index.html) was used to obtain the general information of miR-582-5p. Publicly available tools including TargetScan (http://www.targetscan.org/), miRanda (http://www.microrna.org/), miRDB (http://www.mirdb.org/) and mirWalk (http://zmf.umm.uni-heidelberg.de/apps/zmf/mirwalk2/) were used to predict the candidate target genes of miR-582-5p. And also TransmiR (http://www.cuilab.cn/transmir) was used to identify upstream regulators that may be responsible for miR-582-5p.

### Dual-Luciferase Reporter Assay

The mutant (MUT) or wild-type (WT) sequences containing the predicted target site of miR-582-5p in the 3′UTRs of COL5A1 mRNA were inserted into the mir-GLO vectors (Promega Corporation, Madison, WI, USA). For the luciferase reporter assay, 786-O and Caki-1 cells (1× 10^5^ cells per well) were seeded in 24-well plates and co-transfected with 300 ng of WT or MUT luciferase vector together with30nM of either miR-582-5p mimics or miR-NC. Then the luciferase activity was determined at 48 h after transfection using a Dual-Luciferase^®^ Assay System (Promega) according to the manufacturer’s instructions.

### Chromatin Immunoprecipitation (ChIP)

ChIP analysis was conducted using ChIP assay kit (Millipore, 17-610) based on the manufacturer’s instructions. A total of 1×10^7^–5×10^7^ cells were collected and 1% formaldehyde (Bio-Rad, CA, USA) was used to crosslink the proteins to the DNA for 25 min. Chromatin was sheared to fragments with size of 100–500 bp by sonicating the lysate. After dislodged insoluble substance by centrifugation, 100 μl DNA/protein complexes were taken as input. The samples were incubated with RUNX1 antibody (Cell Signaling Technology), normal rabbit IgG antibodies (Cell Signaling Technology), and protein A/G beads overnight at 4°C. After incubation at 65 °C for 4 h, the crosslinking of input and the samples were reversed. Then, phenol/chloroform (Invitrogen) was involved to recover DNA from the samples. Promoter binding was evaluated *via* PCR with primers of miR-582-5p upstream region.

### Statistical Analysis

All of the experiments were performed independently three times. Results were described as means ± standard deviation (SD). All statistical analysis was performed using GraphPad Prism software version 6.0 (GraphPad, Software, Inc., La Jolla, CA, USA). The correlation between miR-582-5p and COL5A1 expression in RCC tissues was determined using Pearson’s correlation coefficient by GraphPad Prism 6.0 software. Statistical differences were evaluated using Student’s t-test for two-group comparisons and one-way analysis of variance for more than two groups. The p-value of less than 0.05 was considered statistically significant.

## Results

### MiR-582-5p Was Downregulation in ccRCC Tissues and Cell Lines

**Figure 1:**
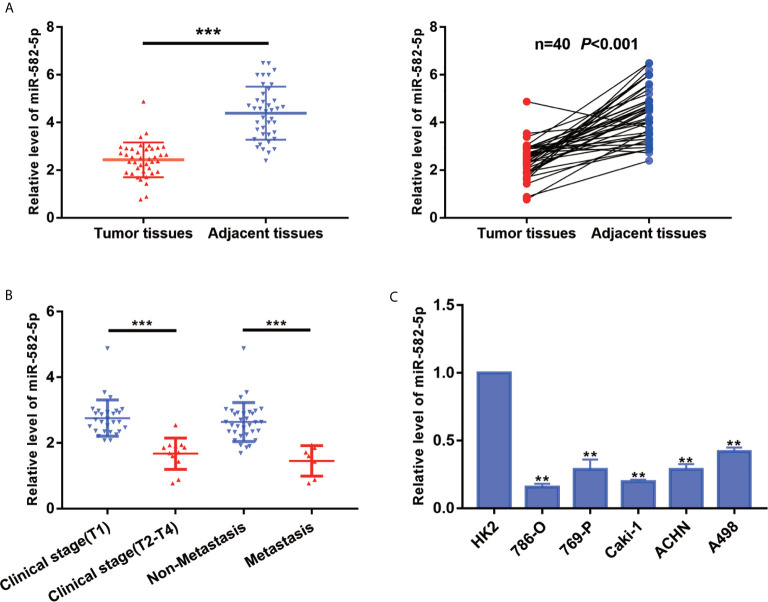
MiR-582-5p was downregulation in ccRCC tissues and cell lines. **(A)** miR-582-5p level in 40 ccRCC tissues and paired adjacent tissues was investigated by qRT-PCR. **(B)** Relative expression of miR-582-5p were determined by qRT-PCR in different clinical stage of ccRCC tissues. **(C)** miR-582-5p level in human normal renal tubular epithelial cell line HK-2 and five ccRCC cell lines was investigated by qRT-PCR; U6 was used as an internal control. The data are shown as the mean ± SD (**P < 0.01; ***P < 0.001). The experiments were repeated three times with similar results.

To explore the primary role of miR-582-5p in ccRCC, we quantified the expression of miR-582-5p in 40 ccRCC tissues and their adjacent normal tissues by qRT-PCR. As compared with normal tissues, the expression level of miR-582-5p was significantly decreased in tumor tissues (*P*<0.001) (**Figure 1A**). Furthermore, all the patients enrolled in the study was divided into two groups according to clinical stage and the status of metastasis respectively. We found that miR-582-5p was high expressed in T1 stage and non-metastatic patients compared with T2-T4 stage and those metastatic patients (**Figure 1B**). As to cell lines, qRT-PCR results showed that miR-582-5p was significantly decreased in various ccRCC cell lines (including 786-O, 769-P, Caki-1, ACHN and A498) compared with human renal tubular epithelial cells (HK2) (**Figure 1C**). In conclusion, miR-582-5p was aberrant shrunk in ccRCC especially in higher T stage and metastatic patients.

### MiR-582-5p Regulated Cell Proliferation and Invasion

**Figure 2:**
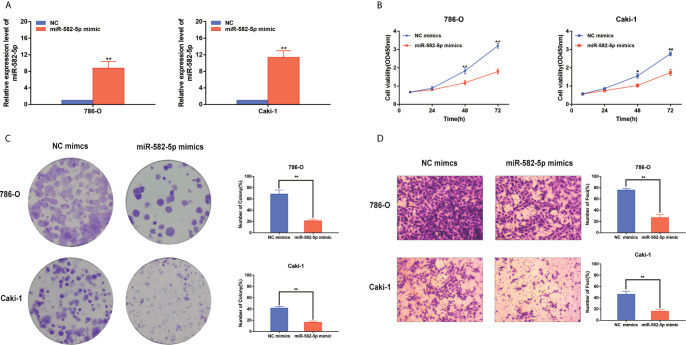
MiR-582-5p regulated cell proliferation and invasion. **(A)** The effect of miR-582-5p mimics was identified by qRT-PCR in 786-O and Caki-1 cells. **(B)** Representative profile of cell growth was examined by CCK-8 assay in 786-O and Caki-1 cells after transfection with miR-582-5p mimics compared with the control. **(C)** Cell proliferation was determined by colony-formation assay of impacts of miR-582-5p in 786-O and Caki-1 cells. **(D)** Representative images revealing the invasion capacities of impacts of miR-582-5p in in 786-O and Caki-1 cells. The data are shown as the mean ± SD (*P < 0.05; **P < 0.01). The experiments were repeated three times with similar results.

Considering that miR-582-5p was obviously attenuated in 786-O and Caki-1 cells, these two cell lined were chosen to be transfected with miR-582-5p mimics and corresponding negative control (NC). The transfection efficiency was verified by qRT-PCR (**Figure 2A**). To investigate the effects of miR-582-5p on cell proliferation, we first conducted CCK8 assay and found that the viability of cells which transfected with miR-582-5p mimics significantly decreased after 72h compared with NC(**Figure 2B**). Furthermore, the results from colony assay reveal that miR-582-5p mimics notably inhibited the colonies (**Figure 2C**). To uncover the role of miR-582-5p in cell invasion, transwell assay was performed. The results displayed that overexpression of miR-582-5p notably weaken the invasive ability of 786-O and Caki-1 cells (**Figure 2D**).

### MiR-582-5p Promoted Xenograft Tumor Formation

**Figure 3:**
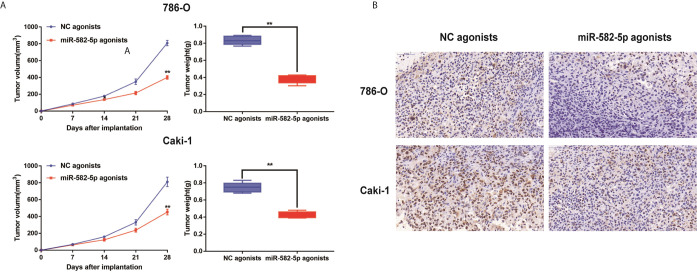
MiR-582-5p promoted xenograft tumor formation. **(A)** Tumor volume and weight were calculated of the xenograft tumors in subcutaneous xenograft mouse model injected with 786-O and Caki-1 cells transfected with NC agonists and miR-582-5p agonists respectively. **(B)** The tumor sections from different transfected groups of xenograft mouse models were subjected to immunohistochemistry staining using antibodies against KLF6 (400×). The data are shown as the mean ± SD (**P < 0.01). The experiments were repeated three times with similar results.

Furthermore, to determine whether miR-582-5p was involved in tumorigenesis *in vivo*, xenograft tumor models were established in BALB/c (nu/nu) mice. MiR-582-5p agonists were used to generate a gain-of-function model since miR-582-5p was down-regulated in 786-O and Caki-1 cells. The volume of the tumors derived from miR-582-5p overexpressing cells was dramatically reduced compared to that in the tumors derived from control cells (**Figure 3A**). Additionally, the IHC result of tissue derived from the mice showed that miR-582-5p obviously decrease the expression Ki-67 protein (**Figure 3B**). In summary, these results demonstrated that miR-582-5p inhibited ccRCC proliferation *in vivo*.

### COL5A1 Was a Potential Target of miR-582-5p

**Figure 4:**
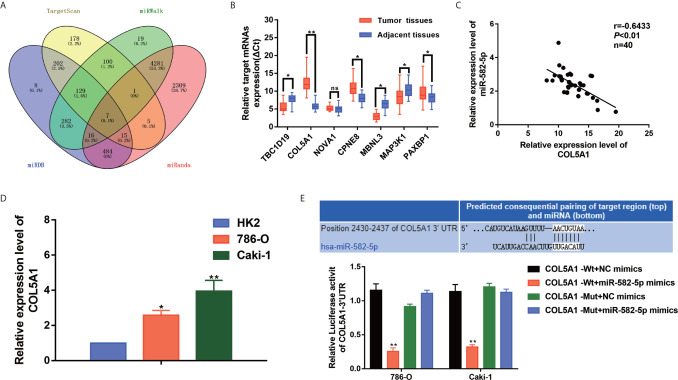
COL5A1 was a potential target of miR-582-5p. **(A)** The candidate gene targets were predicted by intersecting outputs from four distinct prediction algorithms (TargetScan, miRDB, miRWalk, and miRanda). **(B)** qRT-PCR assay confirmed the relative expression of seven candidate target mRNAs of miR-582-5p in 40 paired ccRCC cancer tissues and corresponding paracancerous normal tissues. **(C)** There was an inverse correlation between miR-582-5p and COL5A1. **(D)** COL5A1 mRNA in HK2 cells and ccRCC cell lines (786-O and Caki-1 cells) were investigated by qRT-PCR. **(E)** The potential miR-582-5p seed region at the 3′-UTR of COL5A1 mRNA was computationally predicted. 786-O and Caki-1 cells were co-transfected with miR-582-5p mimics (or NC) with COL5A1-Wt (or COL5A1-Mut) vector. Luciferase activity was normalized by the ratio of firefly and Renilla luciferase signals. The data are shown as the mean ± SD (*P < 0.05; **P < 0.01). The experiments were repeated three times with similar results.

We performed bioinformatics analysis to predict the target genes of miR-582-5p based on four databases including TargetScan, miRWalk, miRDB and miRanda. As shown in **Figure 4A**, 7 candidate mRNAs (TBC1D19, COL5A1, NOVA1, CPNE8, MBNL3, MAP3K1 and PAXBP1) were picked. qRT-PCR were used to further validate the expression level of selected mRNAs in 40 paired clinical specimens. Among these mRNAs, 6 genes were significantly aberrant expressed in tumor tissues compared with their adjacent tissues (3 up-regulated and 3 down-regulated) (**Figure 4B**). Notably, COL5A1 was the most significantly upregulated in the tumor tissues. Further analysis showed that there was a negative correlation between miR-582-5p and COL5A1 in tumor tissues (*r*=0.6433, *P*<0.01) (**Figure 4C**). In addition, we verified the up-regulation expression of COL5A1 in two ccRCC cell lines (786-O and Caki-1) (**Figure 4D**). To further confirm whether COL5A1 was a direct target of miR-582-5p, bioinformatics analysis was performed to search for the presumed miR-582-5p targeting sites in COL5A1 transcripts. It was found that COL5A1 3’-UTR contained a miR-582-5p-binding site (**Figure 4E**). In addition,we constructed luciferase reporter vectors containing the wild type (WT) or mutation type (MuT) fragments of COL5A1 3’-UTR. Then the vectors were co-transfected with miR-582-5p mimics or NC mimics into 786-O and Caki-1 cells. Upregulation of miR-582-5p markedly reduced relative luciferase activity in COL5A1-Wt cells, while the relative luciferase activity in the COL5A1-Mut cells showed no significant difference between the transfected miR-582-5p mimics group and the transfected miR-NC group. Taken together, above results revealed that miR-582-5p could influence COL5A1 expression by directly binding to 3’-UTR of COL5A1.

### Reconstitution of COL5A1 Partially Rescued the miR-582-5p-Mediated Effects

**Figure 5:**
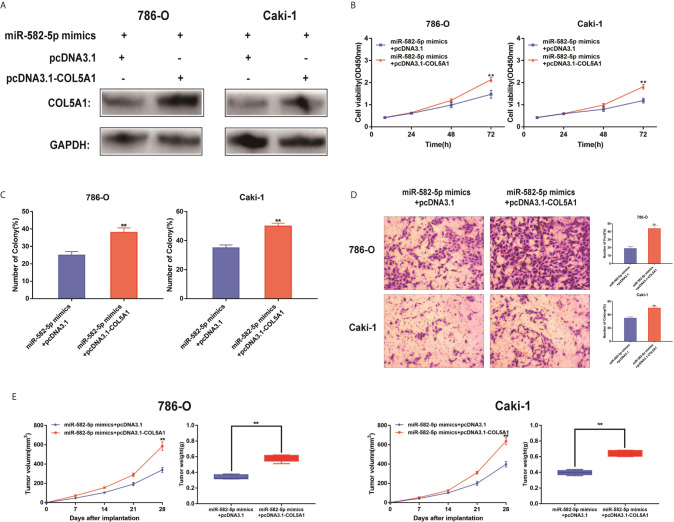
Reconstitution of COL5A1 partially rescued the miR-582-5p-mediated effects. **(A)** Expression levels of COL5A1 in 786-O and Caki-1 cells which co-transfected with miR-582-5p mimics and pcDNA3.1-COL5A1 (or pcDNA3.1) were detected by western blot. GAPDH was used as an internal control. **(B)** CCK-8 assay of 786-O and Caki-1 cells in different transfected groups. **(C)** Cell proliferation was determined by colony-formation assay of different transfected groups in 786-O and Caki-1 cells. **(D)** Representative images revealing the invasion capacities of impacts of different transfected groups in 786-O and Caki-1 cells. **(E)** Tumor volume and weight were calculated of the xenograft tumors in subcutaneous xenograft mouse model injected with 786-O and Caki-1 cells co-transfected with COL5A1 and miR-582-5p mimics or co-transfected with vector and miR-582-5p mimics respectively. The data are shown as the mean ± SD (**P < 0.01). The experiments were repeated three times with similar results.

The functional relevance of COL5A1 targeting by miR-582-5P was investigated by determining whether COL5A1 overexpression could rescue the inhibitory effects of miR-582-5p on ccRCC cell proliferation and invasion. 786-O and Caki-1 cells were co-transfected with miR-582-5p mimics and pcDNA3.1-COL5A1. Western blot analysis validated the COL5A1 protein in the rescue experiments (**Figure 5A**). CCK-8 assay and colony formation assay suggested that the overexpression of COL5A1 rescued the inhibitory effect of miR-582-5p on cell proliferation *in vitro* (**Figures 5B, C**). Transwell assays showed that the invasion ability inhibited by miR-582-5p was remarkably rescued by the COL5A1 overexpression (**Figure 5D**). *In vivo*, the decreased tumor size and weight by miR-582-5p were reversed by COL5A1 overexpression (**Figure 5E**). These results suggested that miR-582-5p influenced the growth and mobility of ccRCC cells by targeting COL5A1.

### RUNX1 Was an Upstream Regulator of miR-582-5p

**Figure 6:**
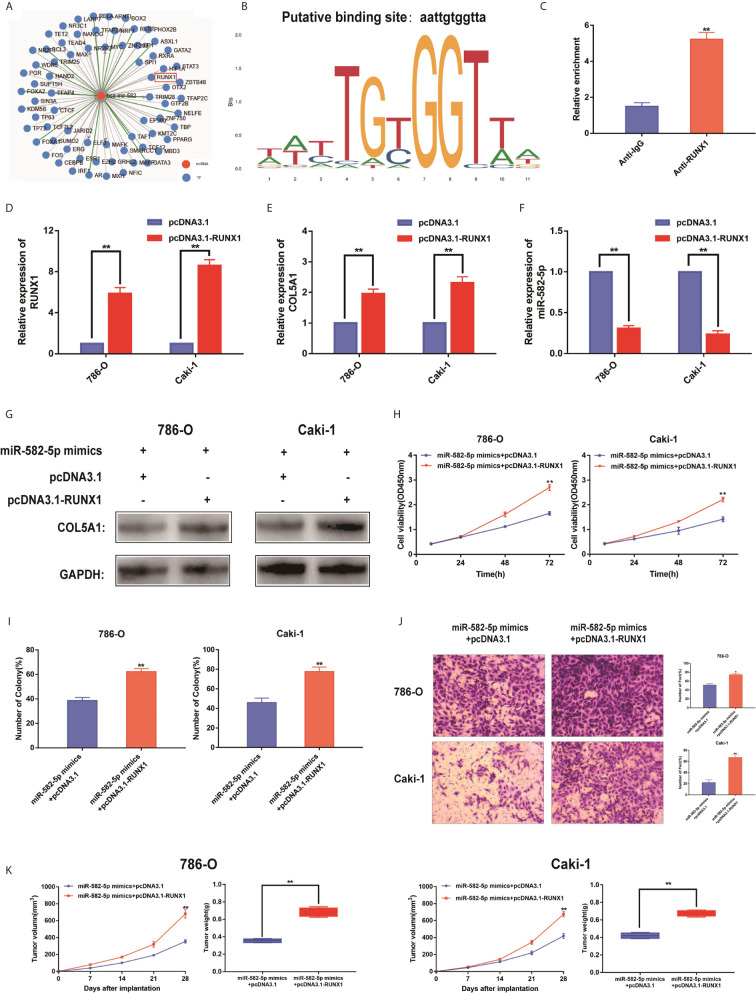
RUNX1 was an upstream regulator of miR-582-5p. **(A)** The candidate upstream regulators were predicted by TransmiR database. **(B)** Based on TransmiR database, the putative score of RUNX1 binding to miR-582-5p was the highest. **(C)** ChIP assay indicated the RUNX1 binds to the putative binding site upstream of miR-582-5p. **(D)** The effect of overexpression RUNX1 by pcDNA3.1 was identified by qRT-PCR. **(E, F)** Levels of miR-582-5p and COL5A1 responding overexpression RUNX1 were detected by qRT-PCR. **(G)** Expression levels of COL5A1 in 786-O and Caki-1 cells which co-trnsfected with miR-582-5p mimics and pcDNA3.1-RUNX1 (or pcDNA3.1) were detected by western blot. GAPDH was used as an internal control. **(H, I)** CCK-8 and colony-formation assays were the influence of 786-O and Caki-1 cells which co-transfected with miR-582-5p mimics and pcDNA3.1-RUNX1 (or pcDNA3.1) on cell proliferation respectively. **(J)** Cell invasion was determined after co-transfected with miR-582-5p mimics and pcDNA3.1-RUNX1 or pcDNA3.1. **(K)** Tumor volume and weight were calculated of the xenograft tumors in subcutaneous xenograft mouse model injected with 786-O and Caki-1 cells co-transfected with RUNX1 and miR-582-5p mimics or co-transfected with vector and miR-582-5p mimics respectively. The data are shown as the mean ± SD (*P < 0.05; **P < 0.01). The experiments were repeated three times with similar results.

To identify upstream regulators that may be responsible for miR-582-5p decrease in ccRCC, based on TransmiR database, we found that numerous genes might be potential upstream regulator of miR-582-5p (**Figure 6A**). Of them, the putative score of RUNX1 binding to miR-582-5p was the highest (**Figure 6B**). Furthermore, ChIP assay indicated that RUNX1 binds to the putative binding site upstream of miR-582-5p (**Figure 6C**). The efficiency of overexpression of RUNX1 was identified by qRT-PCR (**Figure 6D**). Overexpression of RUNX1 led to decreased miR-582-5p and increased COL5A1 expression which indicated that there was an axis among RUNX1/miR-582-5p/COL5A1 signaling (**Figures 6E, F**). According to previous research, we already known that RUNX1 was over-expression in human ccRCC, and high protein expression correlates with poorer survival. Additionally, deletion of RUNXL1 was able to disrupt tumor cell growth both *in vitro* and *in vivo* (24). Subsequently, we recruited a rescue assay to figure out whether miR-582-5p could rescue the overexpressed RUNX1 effect. As shown in **Figure 6G**, after co-transfection of miR-582-5p mimics with pcDNA 3.1-RUNX1 or vector, the expression level of COL5A1 were detected. Moreover, the results showed that miR-582-5p overexpression could partially rescue the effects of RUNX1 on cell proliferation and invasion in ccRCC cell lines (**Figures 6H–J**). *In vivo*, the miR-582-5p induced tumor shrinkage was reversed by RUNX1 overexpression (**Figure 6K**). Overall, the total data indicated that RUNX1 contributed to the tumor growth by modulating miR-582-5p transcription.

## Discussion

**Figure 7:**
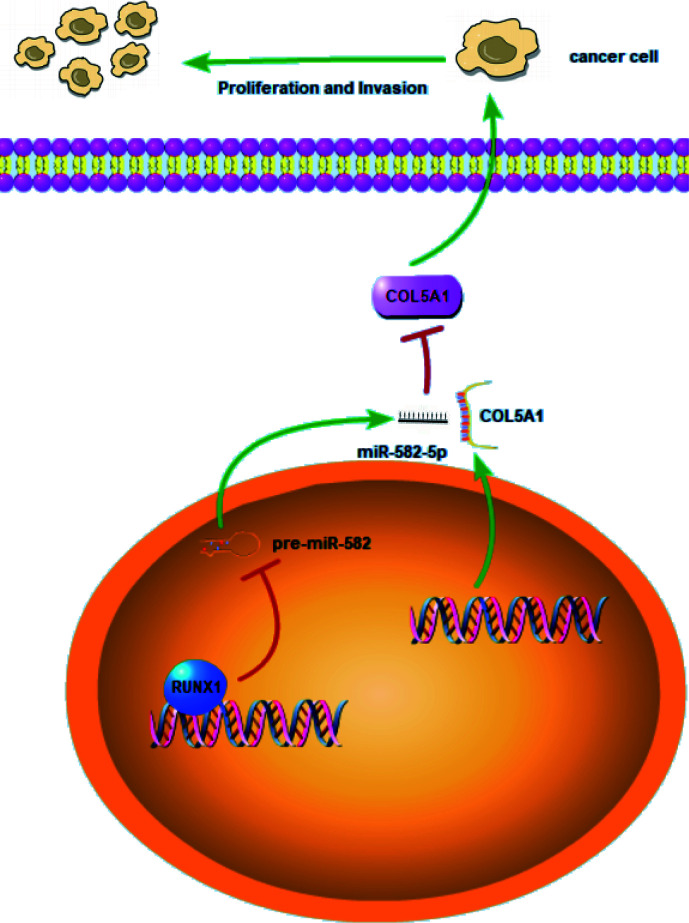
Schematic diagram for miR-582-5p, mediated by RUNX1, regulates cell proliferation and invasion by targeting COL5A1 in ccRCC. RUNX1 was verified to bind to miR-582-5p promoter and attenuated miR-582-5p expression in ccRCC. miR-582-5p regulated cell proliferation and invasion in ccRCC by targeting COL5A1. The experiments were repeated three times with similar results.

RCC is a heterogeneous group of epithelial tumors, among which clear cell RCC (ccRCC) is the most common and accounts for 70–80% of the reported cases of RCC (25). Lack of effective diagnostics in the early stages of the disease, increasing mortality rate, and resistance to therapies in patients with metastatic ccRCC emphasize the need to discover new biomarkers that are applicable for the early diagnosis of ccRCC and detection of metastasis. To this end, many studies have been conduct with the aim of generating molecular markers that regulate RCC progression and metastasis. To our knowledge, many non-coding RNAs including miRNAs play critical role of either oncogene or suppressor in RCC (26). In this study, we discovered a new molecular mechanism related to the proliferation and invasion of ccRCC. As shown in the schematic diagram (**Figure 7**), RUNX1-mediated miR-582-5p regulates the cell proliferation and invasion of ccRCC by targeting COL5A1.

Our study first proved the expression pattern and mechanism of miR-582-5p in ccRCC. Briefly, miR-582-5pexpression was obviously decreased in ccRCC tumor tissues and cells. Moreover, low miR-582-5p expression was related to higher T stage and the status of metastasis in ccRCC patients. Findings also revealed that miR-582-5p overexpression prominently inhibited RCC cell proliferation and invasion capacities. Additionally, cell line-derived xenograft model was utilized which further confirmed the tumor-suppressive role of miR-582-5p in ccRCC. The result was in accordance with previous findings in glioblastoma, prostate cancer, gastric cancer, none-small cell lung cancer, bladder cancer, etc. (13, 14, 16, 21, 22).

As we all know, miRNAs normally regulate biological process by binding to downstream mRNA. Therefore, the identification of target genes of miRNAs plays an important role in the diagnosis and treatment of tumors (9–12). Based on bioinformatic analysis, we found 7 candidate genes among which COL5A1 was the most significant upregulated gene. Collagen type V (COL5) is a regulatory fibril-forming collagen which has at least three different molecular isoforms-COL5A1,COL5A2,COL5A3 (27). Previous evidences show that COL5A1, a member of collagen family protein, was related to extracellular matrix (ECM) which is known as an essential event in metastasis of RCC (28, 29). In fact, COL5A1 is a core gene in metastatic RCC whose expression level correlates with poor survival (30, 31). Previous study by Feng G et al. has found COL5A1 promoted ccRCC cell proliferation, apoptosis, migration, invasion *in vitro* (32). Therefore we presumed that COL5A1 was the target of miR-582-5p. To further confirm this, we constructed a wild-type plasmid and a mutant plasmid (COL5A1-Wt or COL5A1-Mut) of the luciferase reporter vector, and the results showed that luciferase activity in COL5A1-Wt transfected miR-582-5p mimics was significantly lower than the COL5A1-Wt luciferase activity in the transfected miR-NC group. In addition, it was found no significant difference in the luciferase-Mut activity between the transfected miR-582-5p mimics group and the luciferase-Mut activity in the transfected miR-NC group, suggesting that miR-582-5p could inhibit COL5A1 expression. In addition, we then performed rescue assay to confirm the relation between miR-582-5p and COL5A1. The results showed that overexpression of COL5A1 could partially reverse the inhibition of cell proliferation and invasion by miR-582-5p.

In addition, the upstream regulators such as transcription factors may be responsible for dysregulated expression of miRNAs in cancers. By using TransmiR database, we identified RUNX1 as an upstream regulator of miR-582-5p expression. Runt-related transcription factor 1 (RUNX1) is one subunit of the core-binding factors (CBFs) that binds to the core element of many enhancers and promoters (33). RUNX1 was involved in the development of normal hematopoiesis, while its mutation and translocations had been associated with several types of leukemia (34, 35). RUNX1, as an oncogene and anti-oncogene, has also received increasing attention in many solid tumors including breast cancer, gastric cancer and colorectal cancer (36–38). Recent studies have shown that RUNX1 participated into many biological processes by regulating downstream pathway. RUNX1 promoted renal tubular Epithelial-to-Mesenchymal Transition (EMT) and kidney fibrosis by regulating TGF-β (39). In addition, another study has found that RUNX1 suppressed the expression of the miR-17~92 cluster in ovarian cancer (40). Thus, RUNX1 could regulate expression of both protein-coding genes and noncoding genes such as miRNAs in several biological processes. In our study, ChIP assay indicated the RUNX1 bind to the binding site upstream of miR-582-5p. Over-expression of RUNX1 led to decreased miR-582-5p expression and increased COL5A1 expression, which demonstrates that there is an axis among RUNX1/miR-582-5p/COL5A1 signaling. Over-expression of RUNX1 caused increasing cell proliferation and invasion, suggesting that RUNX1 contributed to the tumor growth by modulating miR-582-5p expression.

## Conclusion

In summary, the study availed a better understanding of the function of miR-582-5p in ccRCC. We confirmed a new antionco-miRNA, miR-582-5p, mediated by RUNX1, regulated cell proliferation and invasion by targeting COL5A1 in ccRCC. These findings indicated a tumor suppressor role of miR-582-5p in ccRCC development and might serve as a potential therapeutic target in ccRCC.

## Data Availability Statement

The raw data supporting the conclusions of this article will be made available by the authors without undue reservation.

## Ethics Statement

The studies involving human participants were reviewed and approved by the ethics committee of First Affiliated Hospital of Nanjing Medical University. The patients/participants provided their written informed consent to participate in this study. The animal study was reviewed and approved by the animal ethics committee of Nanjing Medical University. Written informed consent was obtained from the individual(s) for the publication of any potentially identifiable images or data included in this article.

## Author Contributions

JX and ZW: study design and funding support; JX, SZ and FQ: celluar experimnets; SZ and KZ: animal experiments; JX and PC: Data collection and analyses; JX, SZ and KZ: manuscript writing and editing; JY and ZW: supervision. All authors contributed to the work and approved it for publication.

## Conflict of Interest

The authors declare that the research was conducted in the absence of any commercial or financial relationships that could be construed as a potential conflict of interest.
